# Prevalence, phylogeny, and antimicrobial resistance of *Escherichia coli* pathotypes isolated from children less than 5 years old with community acquired- diarrhea in Upper Egypt

**DOI:** 10.1186/s12879-020-05664-6

**Published:** 2020-12-01

**Authors:** Rasha M. M. Khairy, Zahra Atef Fathy, Doaa Mohamed Mahrous, Ebtisam S. Mohamed, Soha S. Abdelrahim

**Affiliations:** 1 Department of Microbiology and Immunology, Faculty of Medicine, Minia University, Minia, 61511 Egypt; 2 Department of Clinical Pathology, Mallawi Hospital, Mallawi, Egypt; 3 Department of Pediatrics, Faculty of Medicine, Minia University, Minia, Egypt

**Keywords:** Diarrheagenic *Escherichia coli* (DEC), Pathotypes- ESBL, AmpC *β*-lactamase

## Abstract

**Background:**

Diarrhoea, affecting children in developing countries, is mainly caused by diarrheagenic *Escherichia coli* (DEC). This study principally aimed to determine the prevalence of DEC pathotypes and Extended-spectrum β-lactamase (ESBL) genes isolated from children under 5 years old with diarrhea.

**Methods:**

A total of 320 diarrhoea stool samples were investigated. *E. coli* isolates were investigated for genes specific for enterotoxigenic *E. coli* (ETEC), enteropathogenic *E. coli* (EPEC), enteroaggregative *E. coli* (EAEC), enteroinvasive *E. coli* (EIEC) and enterohemorrhagic *E. coli* (EHEC) using polymerase chain reaction (PCR). Furthermore, antimicrobial susceptibility testing, detection of antibiotic resistance-genes and phylogenetic typing were performed.

**Results:**

Over all, DEC were isolated from 66/320 (20.6%) of the children with diarrhoea. EAEC was the predominant (47%), followed by typical EPEC (28.8%) and atypical EPEC (16.6%). Co-infection by EPEC and EAEC was detected in (7.6%) of isolates. However, ETEC, EIEC and EHEC were not detected. Phylogroup A (47%) and B2 (43.9%) were the predominant types. Multidrug-resistance (MDR) was found in 55% of DEC isolates. Extended-spectrum *β*-lactamase (ESBL) genes were detected in 24 isolates (24 *blaTEM* and 15 *blaCTX-M*-15). Only one isolate harbored AmpC *β-*lactamase gene (DHA gene).

**Conclusion:**

The study concluded that, EAEC and EPEC are important causative agents of diarrhoea in children under 5 years. MDR among DEC has the potential to be a big concern.

## Background

Diarrhoea is one of the top ten leading causes of death worldwide and the second in low-income countries in children under 5 years old [1]. Diarrheagenic *Escherichia coli* (DEC) is a very important cause of pediatric diarrhoea [2], particularly in developing countries, where these organisms are the main cause of diarrhoea affecting children under 5 years old [3]. The DEC is classified according to specific virulence characters into distinct pathotypes [2]. The epidemiology of the different DEC pathotypes varies according to the geographical variations even within the same area [3–5]. The most common pathotypes in developing countries are enteroaggregative *E. coli* (EAEC), enteropathogenic *E. coli* (EPEC) and enterotoxigenic *E. coli* (ETEC) [5]. The EPEC type is subdivided into typical EPEC (tEPEC), that carries both of the intimin gene (*eae)* and the bundle forming pili (*bfp*) genes, and atypical EPEC (aEPEC) which carries *eae* gene but lacks the *bfp* gene. While the *eae* gene is responsible for attachment and effacement of intestinal epithelial cells, *bfp* gene is encoded for adherence factors [6]. The EAEC, which is an emerging cause of diarrhoea in adults and children worldwide [7], can be determined by the plasmid-encoded gene probe *pCVD432* which explain the aggregative phenotype [8]. Other pathotypes of *E. coli,* that display low levels of incidences such as enterohemorrhagic *E. coli* (EHEC) “a subtype of Shiga toxin-producing *E. coli* (stx1 and stx2)” and enteroinvasive *E. coli* (EIEC), can cause life-threatening diarrhoea [9]. DEC pathotypes can be determined by some techniques, such serotyping, phenotyping and molecular methods [9]. PCR is a highly sensitive and specific assay that gives fast and reliable results [9]. PCR assay can also categorize DEC into different phylogenetic groups [10]. Treatment with antimicrobials is recommended only for severely ill patients. Unfortunately, the treatment of *E. coli* is difficult due to the emergence of antibiotics resistant strains in the last decades [11]. The predominant mechanism of resistance is the hydrolysis of the antibiotic by beta-lactamases. The ability to produce *B*-lactamases, including Extended Spectrum Beta Lactamases (ESBL) and Amp-C *B*-lactamases is frequently acquired through large plasmids holding many different genes resistance (MDR) [12]. Intestinal *E. coli* is one of the important organisms harboring these genes [13]***.*** Information about DEC pathotypes in Egypt is scarce, particularly in south Egypt because molecular techniques are not usually used to determine DEC in medical laboratories. The present study aimed to determine the prevalence, phylogenetic types and resistance patterns of DEC pathotypes among children under 5 years old with acute diarrhoea in Minia, South Egypt.

## Methods

### Study population

The cohort of this study included children less than 5 years of age with acute community-acquired diarrhoea, who visited the Pediatric departments of two major hospitals in Minia, Egypt; Minia university hospital and Minia governorate hospital as outpatients from December 2018 to May 2019. Acute diarrhoea was defined as passage of three or more loose or watery stools per day, plus one or more gastroenteritis symptoms as; nausea, vomiting, abdominal pain, fecal urgency, fever or cramps. Positive rotavirus samples were excluded from the study. Children with a history of antibiotic use in the last 2 weeks were excluded from the study.

### Specimen collection, isolation, and identification of *E. coli*

A total of 320 stool samples were included in the study. One stool sample was collected from each patient in sterile container and transported to the microbiology laboratory in cool box for immediate examination. The samples were examined for consistency, presence of blood, mucous or occult blood. The samples were inoculated into MacConkey broth for enrichment at 37 °C for 24 h. The enrichments were streaked onto MacConkey agar (Oxoid, Basingstoke, United Kingdom). and incubated for 24 h at 37 °C. After that, lactose fermenters isolates were subcultured onto eosin methylene blue agar (EMB) agar (Oxoid, Basingstoke, United Kingdom). Colonies producing greenish metallic sheen on EMB agar were examined by biochemical tests including; IMViC (indole, methyl red, Voges-Proskauer, citrate utilization), urease agar, sugar fermentation and motility tests. In addition the identified *E. coli* isolates were confirmed by chromogenic media (CHROMagar™ Orientation, Paris, France). Samples containing *E. coli* only were included. Hemolytic activity was tested by culturing of isolates on blood agar. *E. coli* isolates were kept in trypticase soy broth (Oxoid, UK) with sterilized 20% glycerol at − 20 °C for further investigations.

### Antimicrobial susceptibility testing

Antimicrobial susceptibility testing was performed using the disk diffusion method and identified according to CLSI guidelines [14]. The used antimicrobial discs were; ceftriaxone (CRO) 30 μg, amoxicillin/clavulanic acid (AMC) 30 μg, ceftazidime (CAZ) 30 μg, meropenem 10 μg (MEM) 10 μg, amikacin (AK) 30 μg, sulphamethoxazole/ trimethoprim (SXT) 300 μg, cefoxitin 30 μg (FOX) and tetracyclin 30 mg (TE) (Thermo Scientific™ Oxoid, UK). Isolates with inhibition zone size ≤22 mm with (CAZ) and ≤ 25 mm with (CRO) were suggested to be ESBL-producers [14]. Double-Disc Synergy Test (DDST) was used for confirmation of ESBL production [15]. In addition, isolates showing an inhibitory zone diameter ≤ 18 mm with (FOX) were suspected to be AmpC *β*-lactamase producers [16]. Carbapenem inactivation method was used for confirmation of carbapenemase production in meropenem- non susceptible isolates [17]. Multiple drug resistance (MDR) was identified as the resistance to at least three different antimicrobial groups [18].

### Molecular identification of diarrheagenic *E. coli* pathotypes

The stored *E.coli* isolates were streaked onto chromogenic media at 37 °C for 24 h. About 3–5 of isolated colonies from each isolate were inoculated onto trypticase soy broth tubes. at 37 °C for 18 h. About 200 μL broth of each isolate was centrifuged (8000 rpm) at 4 °C for 6 mins. The pellet was separated and processed for DNA extraction using GeneJET genomic DNA purification kit according to the manufacturer’s instructions (Thermo scientific**,** USA). For identification of DEC pathotypes, the following virulence genes were investigated using single PCR reactions; *pCVD432* gene for enteroaggregative *E. coli (*EAEC) [19], *eae* gene for both types of enteropathogenic *E.coli* (typical enteropathogenic *E.coli* “tEPEC” and atypical enteropathogenic *E.coli* “aEPEC”) [20], *bfpA* gene for typical enteropathogenic *E.coli* (tEPEC) [21], *LT* gene, *ST* gene for enterotoxigenic *E. coli* (ETEC) [22] and *ipaH gene* for enteroinvasive *E.coli* (EIEC) [23]*.* While (*stx1*and *stx2)* genes for enterohemorrhagic *E. coli* (EHEC) were identified using multiplex PCR [24]. Each single PCR reaction was conducted in a final reaction of 25 μL containing 2 *μ*L of template DNA (approximately 100 ng/*μ*L) and 12.5 μL of master mix (Maxima Hot Start Green PCR Master Mix, Thermo scientific, USA), 10 pmol of each primer and 8.5 *μ*L of nuclease-free water. However the multiplex PCR reaction was performed in a 25 μL reaction mixture containing 3 μL of purified DNA (approximately 100 ng/*μ*L), 12.5 *μ*L of Maxima Hot Start Green PCR Master Mix (Thermo scientific, USA), 1 μL (10 pmol) of each primer (Thermo scientific, USA) and 5.5 μL of nuclease free water. EPEC E2348/69, ETEC H10407, and EAEC positive strain from our laboratory served as the positive control [25].

### Phylogenic analysis

*DEC* isolates were classified into different phylogenic types by triplex PCR using two genes (*chuA* and *yjaA*) and a DNA fragment *TSPE4.C2* as described previously [11].

### Detection of resistance genes

PCR assay was used for identification of resistant genes, *blaTEM, blaSHV* [26] and *blaCTX-M-15* [27]. In addition, a multiplex PCR was done for AmpC *β*-lactamase genes (MOX, FOX, CIT and DHA) genes [28]. PCR assays were performed using Biometra, UNO II thermal cycler (Gottingen, Germany). Primers sequences and amplification parameters used in the study are described in (Table 1). Agarose gel electrophoresis (2%) was used to identify PCR products (Biometra Gottingen, Germany). Positive control was obtained from a previous research [29].

**Table 1:** PCR primers used in the study

Primer	Primers (5_ to 3_)	Amplicon size (bp)	Annealing temperature °C	Reference
*pCVD432*	F-CTGGCGAAAGACTGTATCAT R-CAATGTATAGAAATCCGCTGTT	630	58	[19]
*eae*	F-CTGAACGGCGATTACGCGAA R-CCAGACGATACGATCCAG	917	58	[20]
*bfpA*	F-AATGGTGCTTGCGCTTGCTGC R-GCCGCTTTATCCAACCTGGTA	326	58	[21]
*LT* gene	F-GGCGACAGATTATACCGTGC R-CGGTCTCTATATTCCCTGTT	450	50	[22]
*ST* gene	F-ATTTTTMTTTCTGTATTRTCTT R-CACCCGGTACARGCAGGATT	190	50	[22]
*ipaH*	F-GTTCCTTGACCGCCTTTCCGATACCGTC R-GCCGGTCAGCCACCCTCTGAGAGTAC	600	52	[23]
*stx1*	F-ATAAATCGCCATTCGTTGACTAC R-AGAACGCCCACTGAGATCATC	180	50	[24]
*Stx2*	F-GGCACTGTCTGAAACTGCTCC R-TCGCCAGTTATCTGACATTCTG	255	50	[22]
*ChuA*	F- ACGAACCAACGGTCAGGAT R-TGCCGCCAGTACCAAAGACA	279	59	[10]
*yjaA*	F-TGAAGTGTCAGGAGACGCTG R-ATGGAGAATGCGTTCCTCAAC	211	59	[10]
*TspE4C2*	F-GAGTAATGTCGGGGCATTCA R-CGCGCCAACAAAGTATTACG	154	59	[10]
*blaTEM*	F-AAACGCTGGTGAAAGTA R-AGCGATCTGTCTAT	822	58	[25]
*blaSHV*	F-ATGCGTTATATTCGCCTGTG R-TGCTTTGTTATTCGGGCCAA	753	60	[25]
*CTX-M-15*	F-CACACGTGGAATTTAGGGACT R-GCCGTCTAAGGCCATAAACA	996	55	[26]
*MOX*	F-GCTGCTCAAGGAGCACAGGAT R-CAC ATT GAC ATA GGT GTG GTG C	520		
*FOX*	F-AAC ATG GGG TAT CAG GGA GAT G R-CAA AGC GCG TAA CCG GAT TGG	190	64	[27]
*DHA*	F-AAC TTT CAC AGG TGT GCT GGG T R-CCG TAC GCA TAC TGG CTT TGC	405		
*CIT*	F-TGG CCA GAA CTG ACA GGC AAA R-TTT CTC CTG AAC GTG GCT GGC	462		

### Statistical analysis

Demographic, clinical and laboratory data of the patients were analyzed using SPSS program for windows version 20.0 (IBM, USA). Categorical variables were analyzed using the chi-square test. *P*-values of < 0.05 were considered statistically significant.

## Results

### Demographic and clinical features of the study participants

Overall, 66 (20.6%) of isolates were identified as DEC among children with acute diarrhoea. Of these, 16/66 (24.2%) was aged< 1 year 22/66 (33.4%) of them were between 1 and 2 years old and 28/66 (42.4%) of them aged between 2 and 5 years. Clinical manifestations showed that, 18 (27.3%) of the cases pass more than 3 loose stools daily, while 48(72.7%) pass more than 5 loose stools daily. Presence of mucus and blood were reported in 58 (87.8%) and 14 (21.2%) of samples respectively. Vomiting was detected in 29 (43.9%) of cases and hemolytic activity was detected in 10 (15.2%) of cases.

### The detected pathotypes in DEC strains

**Fig. 1:**
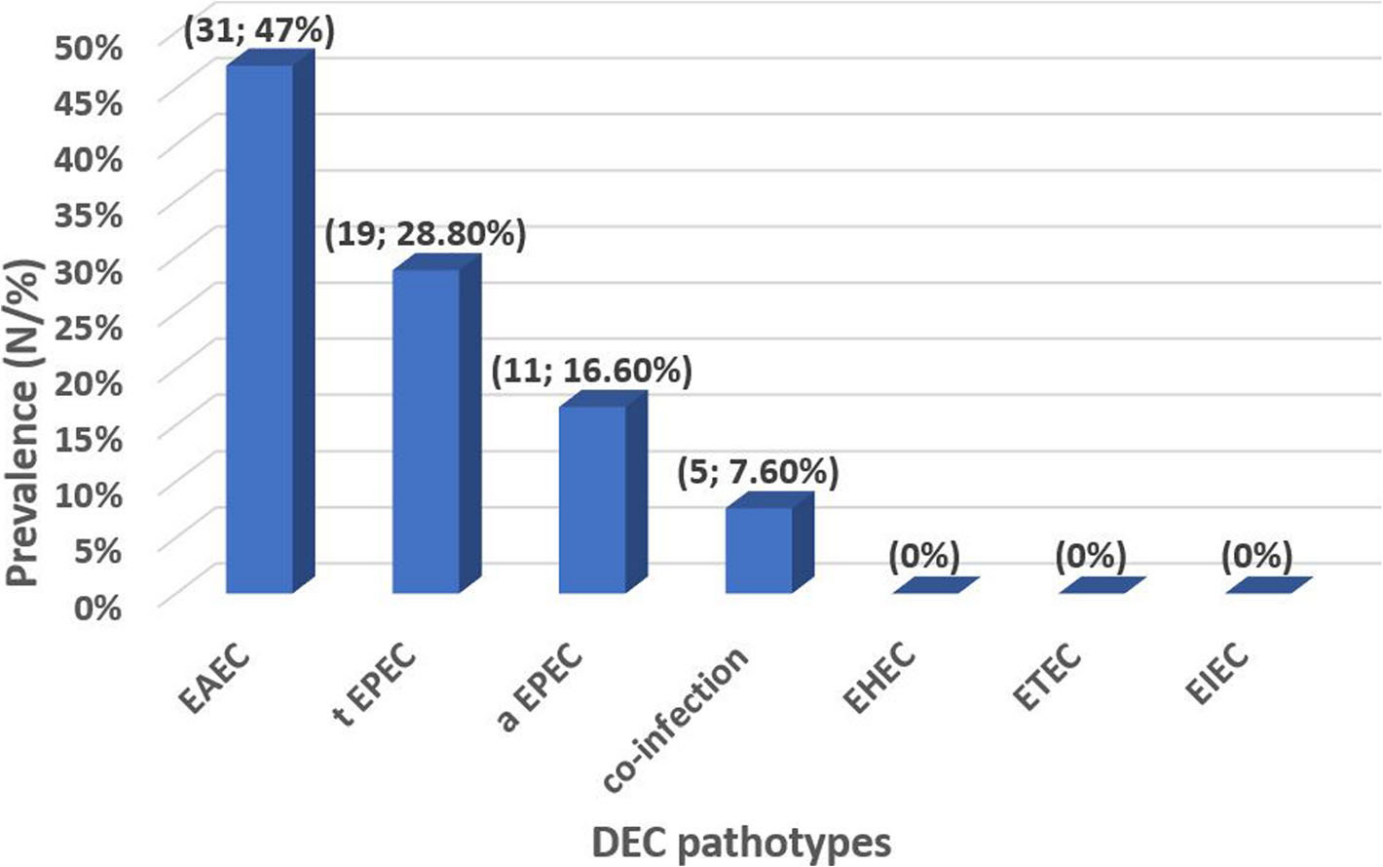
Prevalence of DEC detected in stool samples. DEC: diarrheagenic *E. coli;* EAEC: enteroaggregative *E. coli* tEPEC; typical enteropathogenic *E*. *coli*, aEPEC; A typical enteropathogenic *E. coli;* EIEC: enteroinvasive *E. coli;* EHEC: enterohemorrhagic *E. coli;* ETEC: enterotoxigenic *E. coli*

**Fig. 2:**
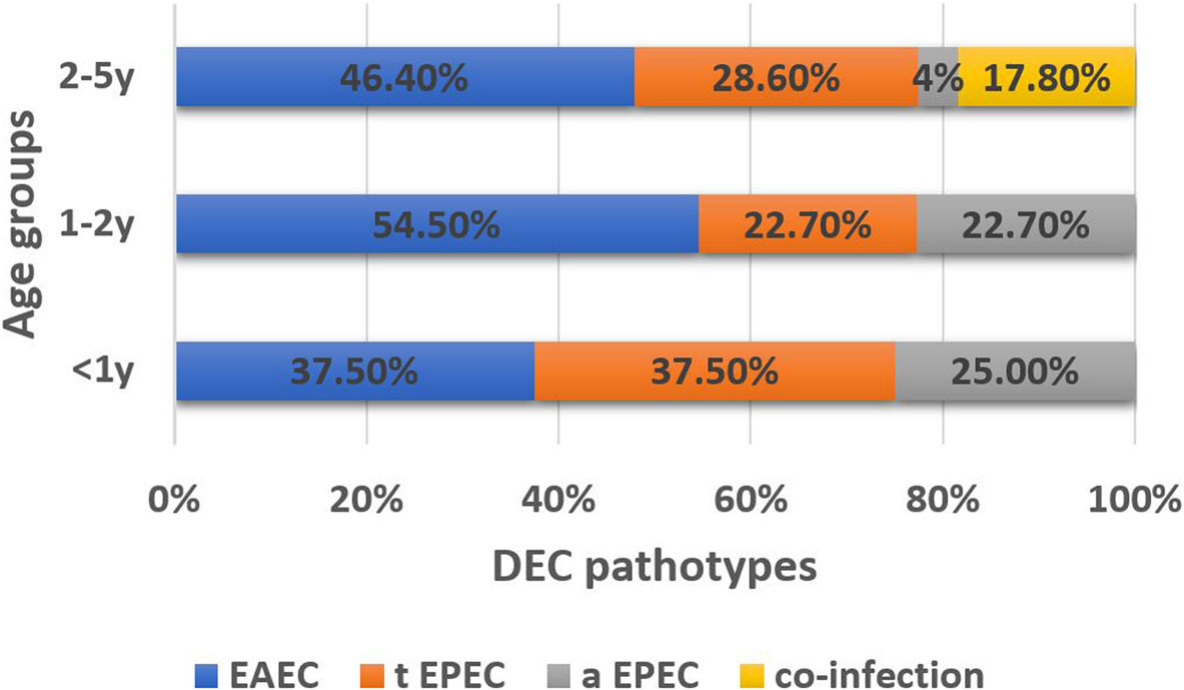
The distribution of DEC types among age groups. EAEC; enteroaggregative *E. coli*, tEPEC; typical enteropathogenic *E. coli*, aEPEC; A typical enteropathogenic *E. coli.* The proportion of EAEC and aEPEC were significantly higher in children under 2 years (*p* = 0.046 and 0.05, respectively)

In the current study, 31/66 (47%) of isolates were identified as EAEC (positive *pCVD432* gene), 19/66 (28.8%) of isolates were identified as tEPEC (positive *eae* gene*+ bfpA* gene) and 11/66 (16.6%) of isolates were identified as aEPEC (positive *eae* gene only). However, 5/66 (7.6%) of isolates had co-infection (4 isolates contain tEPEC + EAEC, while 1 isolate contains aEPEC + EAEC). EHEC, ETEC and EIEC were not detected in the study isolates (Fig. 1). Distribution of DEC types among age groups are presented in (Fig. 2) and the distribution of the clinical manifestations among the DEC pathotypes are summarized in (Table 2).

**Table 2:** Distribution of the clinical manifestations among the DEC pathotypes

Clinical data	EAEC (*N* = 31)	tEPEC (*N* = 19)	aEPEC (*N* = 11)	Co-infection (*N* = 5)	*p*-value
Diarrhoea> 3/day	1 (3.2%)	4 (21%)	11 (100%)	2 (40%)	0.05
Diarrhoea> 5/day	30 (96.7%)	15 (78.9%)	0 (0%)	3 (60%)	0.08
Vomiting	8 (25.8%)	9 (47.3%)	7 (63.6%)	5 (100%)	0.27
Blood	10 (32.2%)	0 (0%)	1 (9.1%)	3 (60%)	0.21
Mucus	23 (74.2%)	19 (100%)	11 (100%)	5 (100%)	0.88
Hemolytic activity	4 (12.9%)	1 (5.2%)	3 (27.2%)	2 (40%)	0.27

*EAEC* enteroaggregative *E. coli*, *tEPEC* typical enteropathogenic *E. coli*, *aEPEC* A typical enteropathogenic *E. coli*

### Distribution of phylogenetic groups among DEC isolates

**Fig. 3:**
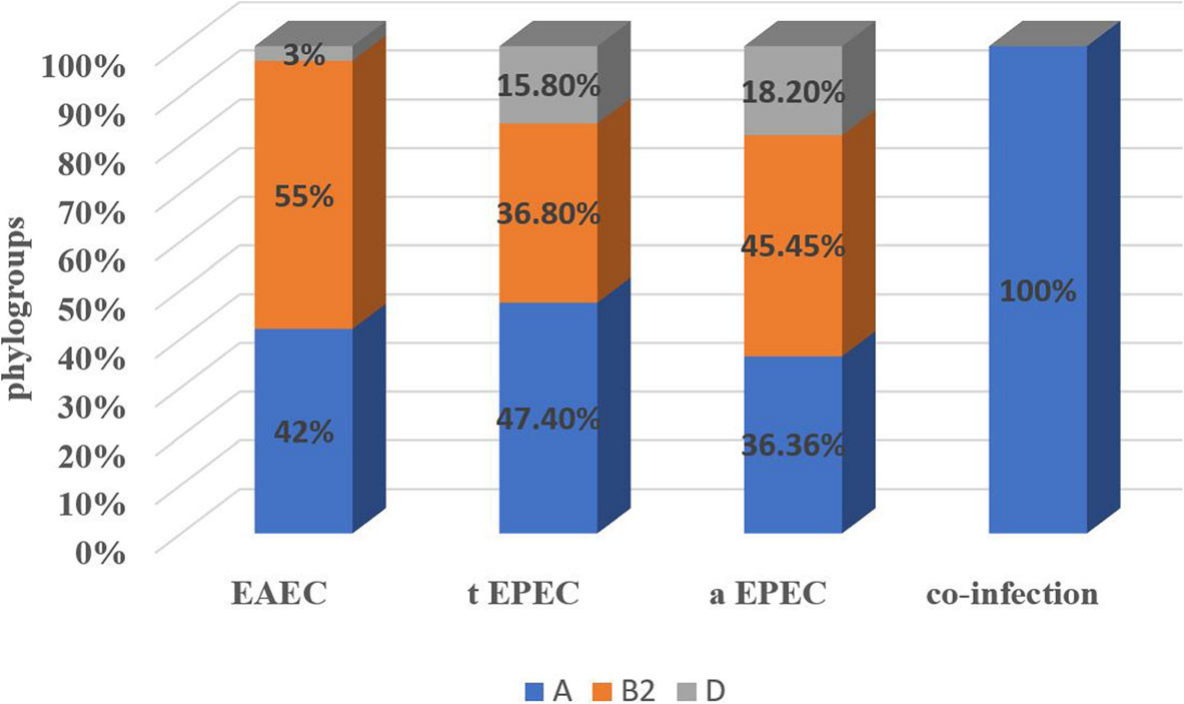
The distribution of phylogenetic groups among DEC types. EAEC; enteroaggregative *E. coli*, tEPEC; typical enteropathogenic *E. coli*, aEPEC; A typical enteropathogenic *E. coli*

The distribution of phylogenetic groups among the DEC isolates reveled the predominance of group A (31/66, 47%) followed by group B2 (29/66, 43.9%) then group D (6/66, 9.1%). However, group B1 was not detected. The distribution of phylogenetic groups among DEC types are presented in (Fig. 3).

### Antimicrobial resistance of DEC isolates

Antibiotic resistance rates of the study isolates were as follows; 22.7, 24.2, 60.6, 62.1, 66.6%, 72.7, and 77.3%, of isolates were resistant to cefoxitin, amikacin, amoxicillin-clavulanate, trimethoprim-sulphamethoxazole, ceftriaxone, ceftazidime, and tetracycline respectively. On the other hand, resistance to meropenem was only (9.1%). Multiple drug resistance (MDR) was detected in 55% of isolates. ESBL production was identified using DDST in (60.6%) of isolates. Carbapenemase production was not detected. Antimicrobial susceptibility among different DEC types are presented in (Table 3).

**Table 3:** Antimicrobial sensitivity patterns among different *E. coli* types

Antibiotics		***E coli*** types				
		EAEC ***N*** = 31	t EPEC ***N*** = 19	a EPEC ***N*** = 11	Co-infection ***N*** = 5	Total ***N*** = 66
**MEM**	Sensitive	27 (87.10%)	16 (84.21%)	10 (90.9%)	5 (100%)	58 (87.9%)
	Intermediate	2 (6.45%)	0 (0.0%)	0(0.0%)	0 (0.0%)	2 (3%)
	Resistant	2 (6.45%)	3 (15.79%)	1 (9.09%)	0 (0.0%)	6 (9.1%)
**CAZ**	Sensitive	4 (12.90%)	3 (15.79%)	0 (0.0%)	0 (0.0%)	7 (10.6%)
	Intermediate	6 (19.36%)	4 (21.05%)	1 (9.09%)	0 (0.0%)	11 (16.6%)
	Resistant	21 (67.74%)	12 (63.16%)	10 (90.9%)	5 (100.0%)	48 (72.7%)
**CRO**	Sensitive	7 (22.58%)	4 (21.05%)	0 (0.0%)	1 (20.0%)	12 (18.2%)
	Intermediate	8 (25.81%)	2 (10.53%)	0 (0.0%)	0 (0.0%)	10 (15.2%)
	Resistant	16 (51.61%)	13 (68.42%)	11 (100.0%)	4 (80.0%)	44 (66.6%)
**FOX**	Sensitive	21 (67.74%)	10 (52.63%)	4 (36.36%)	4 (80.0%)	39 (59.1%)
	Intermediate	4 (12.91%)	5 (26.32%)	3 (27.28%)	0 (00.0%)	12 (18.2%)
	Resistant	6 (19.35%)	4 (21.05%)	4 (36.36%)	1 (20.0%)	15 (22.7%)
**AK**	Sensitive	13 (41.93%)	10 (52.63%)	8 (72.73%)	4 (80.0%)	35 (53%)
	Intermediate	8 (25.81%)	5 (26.32%)	1 (9.09%)	1 (20.0%)	15 (22.7%)
	Resistant	10 (32.26%)	4 (21.05%)	2 (18.18%)	0 (00.0%)	16 (24.2%)
**SXT**	Sensitive	7 (22.58%)	6 (31.58%)	0 (00.0%)	0 (0.0%)	13 (19.7%)
	Intermediate	3 (9.68%)	4 (21.05%)	4 (36.36%)	1 (20.0%)	12 (18.2%)
	Resistant	21 (67.74%)	9 (47.37%)	7 (63.64%)	4 (80.0%)	41 (62.1%)
**TE**	Sensitive	3 (9.68%)	5 (26.32%)	0 (00.0%)	4 (80.0%)	12 (19.2%)
	Intermediate	0 (00.0%)	3 (15.79%)	0 (00.0%)	0 (00.0%)	3 (4.5%)
	Resistant	28 (90.32%)	11(57.89%)	11 (100.0%)	1 (20.0%)	51 (77.3%)
**AMC**	Sensitive	5 (16.13%)	2 (10.53%)	1 (9.09%)	0 (00.0%)	8 (12.1%)
	Intermediate	1 (3.22%)	5 (26.32%)	7 (63.64%)	5 (100.0%)	18 (27.3%)
	Resistant	25 (80.65%)	12 (63.15%)	3 (27.27%)	0 (00.0%)	40 (60.6%)

sulfamethoxazole -Trimethoprim, (SXT); Amikacin (AK); Cefoxitin (FOX); Ceftriaxone (CRO); Meropenem (MEM); Tetracycline (TE); Ceftazidime (CAZ); Amoxicillin-clavulanate (AMC)

### *Β*-lactamases identification

ESBL production was identified phenotypically in 40/66 (60.6%) of isolates, however molecular examination of ESBL genes revealed that only 24 isolates harbored genes. *blaTEM* gene was detected in 24/40 (60%), and *blaCTX-M-15* was detected in 15/40 (37.5%) of ESBL-producers. Co-carriage of *TEM* and *CTX-M-15* genes were occurred in 15/40 (37.5%), however none of the isolates was carrying *SHV* gene. Multiplex PCR was performed to detect AmpC *β*-lactamase genes in cefoxitin resistant isolates (15). Only one of them harbored one gene of AmpC *β-*lactamase genes (DHA gene) 1/15 (6.6%). The distribution of resistance genes among DEC types are presented in (Table 4).

**Table 4:** The distribution of resistance genes among DEC types

*E. coli* types	N	*TEM* gene	*SHV* gene	*CTX-M-15* gene	AmpC genes
DEC	66	24 (36.4%)	–	15 (22.7%)	–
EAEC	31	13 (41.9%)	–	7 (22.6%)	–
tEPEC	19	2 (10.5%)	–	2 (10.5%)	–
aEPEC	11	4 (36.4%)	–	3 (27.2%)	–
Co-infection	5	5 (100%)	–	3 (60%)	1 (20%) DHA gene

*EAEC* enteroaggregative *E. coli*, *tEPEC* typical enteropathogenic *E*. *coli*, *aEPEC* A typical enteropathogenic *E. coli*

## Discussion

Diarrheagenic *E. coli* (DEC (pathotypes are responsible for most of acute diarrhoeal episodes in children < 5 years old in developing countries [3]. The travel of persons from one country to another or even within the same region increases the risk of DEC transmission. So, knowledge about the epidemiology of these infections in each region or country is very important for health policy makers [30]. Data about DEC incidences from Egypt and North Africa using molecular methods is scarce. Therefore, the findings of the present study may facilitate further epidemiological and therapeutic prospects. DEC prevalence in this study (20.6%) agrees with other studies elsewhere [3, 5]. However lower prevalence was also reported in Nigeria (12.8%) [31] and Libya (8.6%) [4]. The prevalence of DEC among children aged< 1 year, aged 1–2 years and aged 2–5 years old were 24.2, 33.4 and 42.4% respectively. These findings agree with Zhou et al., [32], who reported that (52.0%) of cases were less than 2 years old and 48% aged from 2 to 5 years old [32]. EAEC was the most frequent isolated pathotype in the present study (47%) followed by EPEC, that agrees with several previous studies in other developing countries [3, 4, 30, 33, 34]. For many decades, tEPEC was more common than aEPEC, particularly in poor areas [35], however, the epidemiology of EPEC infections has shifted. In the last decade, aEPEC has become more frequent in high-income and also in developing countries [36, 37]. Less studies still report tEPEC as more prevalent than aEPEC and as an important cause of diarrhoea [32, 38], that agrees with our results where (28.8%) of isolates were identified as tEPEC and (16.6%) of isolates were identified as aEPEC. Occurrence of co-infection was also found in our study, where (5%) of isolates had co-infection (4 isolates “tEPEC + EAEC” and 1 isolate “aEPEC + EAEC”). Isolation of multiple enteric pathogens from the same patient in developing countries is not rare, thus, co-infection was also detected previously in several countries [32, 38].

Although ETEC was detected in several previous studies [3, 34], our study could not detect it. However, our finding is comparable with some previous studies in Egypt and other African countries, which reported that ETEC was poorly detected (3.2%) [30, 34]. EHEC and EIEC were also not detected in the current study that agrees with several previous studies in Egypt and other countries [34, 3, 4]. Several factors may lead to such differences in the prevalence rates of DEC pathotypes found in the present and previous studies including geographical locations, study subjects, and standard of sanitation. The incidence of different DEC pathotypes was not uniform in all age groups of the current study. EPEC was more common in children under 2 years old, while EAEC was more in children aged from 2 to 5 years old. This is consistent with previous studies reported that, EPEC is among the most important pathogens infecting children under 2 years old in the developing countries [3, 39]. The distribution of phylogenetic groups among the studied DEC pathotypes revealed the predominance of group A (47%) followed by B2 (43. 9%) then D (9.1%), however, group B1was not present among the study isolates. Phylogenetic group B2 was the most frequent among EAEC strains, that disagrees with a previous Egyptian study recorded group D as the predominant group in DEC and also in EAEC strains [37]. In EPEC strains of the present study, phylogroup A was the commonest followed by B2 and lastly group D, that disagrees with Bozcal et al., [40], who reported group D as the most prevalent group among EPEC strains [40]. Okeke et al., [41], reported that EAEC strains were mostly belonging to phylogenetic groups A, B1, D and only 4.7% of them belonged to group B2 [41]. This difference in phylogenetic groups among our EAEC isolates and other studies could be due to different ancestral origins of EAEC in each area. DEC strains can be reservoirs for antibiotic resistance genes [42]. Multiple drug resistance was detected in 51% of the current study isolates. The observed resistance might be a result of antimicrobials abuse, which is common in low-income countries, including Egypt [43]. Supporting this hypothesis, most of strains in the current study were resistant to the commonly used antibiotics in the study area, such as 3rd generation cephalosporins, that agrees with several previous studies [44]. Resistance to 3rd generation cephalosporins is caused mainly by ESBL production. In the current study ESBL production was identified phenotypically in 40/66 (60.6%) of DEC isolates, however molecular examination of ESBL genes revealed that *blaTEM* gene was detected in 24/66(36.3%), and *blaCTX-M-15* was detected in 15/66 (22.7%) of DEC isolates. Co-carriage of *blaTEM* and *blaCTX-M-15* genes was detected in 15/66 (22.7%). The frequencies of both genes in different pathotypes were similar. However, none of the isolates was carrying *SHV* gene. These findings disagree with Khoshvaght et al., [45], who reported high frequencies of *blaTEM and blaCTX-M* genes in EAEC isolates (78.9 and 63.1% respectively) [45]. Our study disagrees also with Ali et al., who reported that, more than 78% of EAEC and none of the EPEC isolates harbored ESBL genes [34]. However, Zhou et al., have reported a very high percentage (93.3%) of positive *ESBL* genes [32]. Out of cefoxitin resistant isolates, only one AmpC *β*-lactamase gene (DHA gene) was detected 1/15 (6.6%), that was higher than a previous report in Egypt [46].

## Conclusion

This study provides updated data about the prevalence of DEC Pathotypes, which can help future epidemiological studies on DEC in Egypt and North Africa. The current findings can also help in setting appropriate diagnostic and therapeutic strategies against DEC pathotypes. EAEC and EPEC are important causative agents of diarrhoea in young Egyptian children, with the majority of isolates belong to phylogenetic groups A and B2. The emergence of Multiple-drug resistance among DEC strains has the potential to be a big public health concern in Egypt, particularly ESBL producers. *BlaTEM* and *blaCTX-M-15* were the major genetic determinants among ESBL producing DEC strains. A very few reports have evaluated the importance of antibiotics for the treatment of DEC; however, our findings highlight the importance of continuous assessment of the resistance profile for the suitable selection of antibiotics.

## Data Availability

All data generated or analyzed during this study are included in this article and supplementary file.

## Abbreviations

aEPEC: A typical enteropathogenic *E. coli*
*bfpA*: Bundle-forming pilus major subunit
DEC: Diarrheagenic *Escherichia coli*
EAEC: Enteroaggregative *E. coli*
EHEC: Enterohemorrhagic *E. coli*
EIEC: Enteroinvasive *E. coli*
EPEC: Enteropathogenic *E. coli*
ESBL: Extended-spectrum beta-lactamase
ETEC: Enterotoxigenic *E. coli*
LT: Heat labile
ST: Heat stable
*Stx*: Shiga toxin
tEPEC: Typical enteropathogenic *E. coli*
